# Associations of Menstrual Cycle Characteristics Across the Reproductive Life Span and Lifestyle Factors With Risk of Type 2 Diabetes

**DOI:** 10.1001/jamanetworkopen.2020.27928

**Published:** 2020-12-21

**Authors:** Yi-Xin Wang, Zhilei Shan, Mariel Arvizu, An Pan, JoAnn E. Manson, Stacey A. Missmer, Qi Sun, Jorge E. Chavarro

**Affiliations:** 1 Department of Nutrition, Harvard T.H. Chan School of Public Health, Boston, Massachusetts; 2 Department of Epidemiology and Biostatistics, School of Public Health, Tongji Medical College, Huazhong University of Science and Technology, Wuhan, Hubei, PR China; 3 Department of Epidemiology, Harvard T.H. Chan School of Public Health, Boston, Massachusetts; 4 Channing Division of Network Medicine, Department of Medicine, Brigham and Women’s Hospital, Harvard Medical School, Boston, Massachusetts; 5 Division of Preventive Medicine, Department of Medicine, Brigham and Women’s Hospital, Harvard Medical School, Boston, Massachusetts; 6 Department of Obstetrics and Gynecology and Reproductive Biology, College of Human Medicine, Michigan State University, Grand Rapids

## Abstract

**Importance:**

Menstrual cycle dysfunction is associated with insulin resistance, a key feature early in the pathogenesis of type 2 diabetes. However, the evidence linking irregular and long menstrual cycles with type 2 diabetes is scarce and inconsistent.

**Objectives:**

To evaluate the associations between menstrual cycle characteristics at different points throughout a woman’s reproductive life span and risk of type 2 diabetes and the extent to which this association is modified by lifestyle factors.

**Design, Setting, and Participants:**

This prospective cohort study included 75 546 premenopausal US female nurses participating in the Nurses’ Health Study II from 1993 to June 30, 2017. Data analysis was performed from February 1 to December 30, 2019.

**Exposures:**

Self-reported usual length and regularity of menstrual cycles at the age ranges of 14 to 17 years, 18 to 22 years, and 29 to 46 years.

**Main Outcomes and Measures:**

Incident type 2 diabetes identified through self-report and confirmed by validated supplemental questionnaires.

**Results:**

Among the 75 546 women in the study at baseline, the mean (SD) age was 37.9 (4.6) years (range, 29.0-46.0 years). A total of 5608 participants (7.4%) had documented new cases of type 2 diabetes during 1 639 485 person-years of follow-up. After adjustment for potential confounders, women reporting always having irregular menstrual cycles between the age ranges of 14 to 17 years, 18 to 22 years, and 29 to 46 years were, respectively, 32% (95% CI, 22%-44%), 41% (95% CI, 23%-62%), and 66% (95% CI, 49%-84%) more likely to develop type 2 diabetes than women reporting very regular cycles (within 3-4 days of expected period) in the same age range. Similarly, women reporting a usual cycle length of 40 days or more between the age ranges of 18 to 22 years and 29 to 46 years were, respectively, 37% (95% CI, 19%-57%) and 50% (95% CI, 36%-65%) more likely to develop type 2 diabetes during follow-up compared with women reporting a usual cycle length of 26 to 31 days in the same age ranges. These associations appeared to be stronger among women with overweight or obesity, a low-quality diet, and low levels of physical activity. The relative excess risk of type 2 diabetes due to the interaction between irregular and long menstrual cycles and the overall unhealthy lifestyle score was 0.73 (95% CI, 0.57-0.89) and 0.68 (95% CI, 0.54-0.83), respectively.

**Conclusions and Relevance:**

In this cohort study of US female nurses participating in the Nurses’ Health Study II, irregular and long menstrual cycles throughout life were associated with a greater risk of type 2 diabetes, particularly among women with overweight or obesity, a low-quality diet, and low levels of physical activity.

## Key Points

**Question:**

Are irregular or long menstrual cycles and unhealthy lifestyle factors jointly associated with type 2 diabetes?

**Findings:**

In this prospective cohort study of 75 546 women, irregular and long menstrual cycles across the reproductive life span were associated with a greater risk of type 2 diabetes. These associations were stronger among women with overweight or obesity, low-quality diet, and low levels of physical activity.

**Meaning:**

These findings suggest that menstrual cycle characteristics may serve as an early sign of the long-term risk of developing type 2 diabetes and that lifestyle interventions may be a useful strategy to reduce the risk of type 2 diabetes among women with menstrual cycle dysfunction.

## Teaser

This cohort study evaluates the associations between menstrual cycle characteristics at different points throughout a woman’s reproductive life span and the risk of type 2 diabetes and the extent to which this association is modified by lifestyle factors.

## Introduction

Diabetes is a global epidemic that, according to the International Diabetes Federation, affected 463 million adults aged 20 to 79 years in 2019 and is expected to increase by 51% to 700 million adults by 2045.^1^ The prevalence of diabetes during 2019 was similar among women and men (9.0% vs 9.6%); by 2045, 11.1% of women worldwide will have diabetes.^1^ Type 2 diabetes is the most common type of diabetes, and its associated complications have life-threatening health effects,^2^ making it important to identify groups with increased susceptibility and develop strategies to promote prevention.

Irregular and long menstrual cycles are common endocrine disorders among women of reproductive age, with an estimated prevalence of nearly 20%.^3^ Many studies have shown that menstrual cycle dysfunction is associated with insulin resistance,^4^ a key feature early in the pathogenesis of type 2 diabetes.^5^ However, evidence linking irregular or long menstrual cycles with type 2 diabetes is scarce and inconsistent.^6,7,8,9^ In addition, no study has assessed whether the same phenotype across different stages of the reproductive life span has a similar association with the risk of type 2 diabetes. More important, while compelling evidence from observational studies and clinical trials has shown that overweight and lifestyle factors, such as smoking, diet, and physical activity, are important factors associated with type 2 diabetes,^10^ it is still unclear whether these factors can modify the association between menstrual cycle characteristics and type 2 diabetes. Therefore, we explored the associations between menstrual cycle characteristics at different points throughout a woman’s reproductive life span and risk of type 2 diabetes among female nurses participating in the Nurses’ Health Study II and evaluated whether the associations were modified by overweight and lifestyle factors.

## Methods

### Study Population

The Nurses’ Health Study II is an ongoing prospective cohort study established in 1989 by recruiting 116 429 US female nurses aged between 25 and 42 years.^11^ Women are followed up biennially via postal or electronic questionnaires. The follow-up response rate of each cycle exceeds 90%. Returning completed questionnaires is considered as evidence of informed consent. Study procedures have been approved by the institutional review boards of Brigham and Women’s Hospital and the Harvard T.H. Chan School of Public Health. This study followed the Strengthening the Reporting of Observational Studies in Epidemiology (STROBE) reporting guideline.

In the 1989 questionnaire, participants retrospectively reported their menstrual cycle characteristics during high school (aged 14-17 years) and at ages 18 to 22 years. In the 1993 follow-up questionnaire when participants were aged 29 to 46 years, they reported their current usual menstrual cycle length and regularity. Women were excluded if they had died or received a diagnosis of cancer, diabetes, coronary heart disease, or stroke (n = 5734) or had reached menopause by 1993 (n = 4865). We also excluded women who did not report menstrual cycle characteristics in the 1989 and 1993 questionnaires (n = 27 442); had missing data on date of birth (n = 17); had missing data on smoking, body mass index (BMI; calculated as weight in kilograms divided by height in meters squared), diet, or physical activity (n = 2539); or never returned follow-up questionnaires (n = 286). After exclusions, 75 546 premenopausal women were followed up from 1993 to June 30, 2017. Age-standardized characteristics were similar between included and excluded women owing to missing data on exposure and lifestyle (eTable 1 in the Supplement).

### Menstrual Cycle Characteristics

Participants reported characteristics of their menstrual cycles by regularity and length, excluding periods of pregnancy or lactation. Cycle regularity was reported as very regular (within 3-4 days of expected period), regular (within 5-7 days of expected period), usually irregular, always irregular, or no period. Usual cycle length was reported as less than 21 days, 21 to 25 days, 26 to 31 days, 32 to 39 days, 40 to 50 days, or more than 50 days or too irregular to estimate. Self-reports of menstrual cycle characteristics have been documented to be reliable in other studies.^3,12^ In this cohort of women who reported regular cycles between the ages of 18 and 22 years, 10 278 of 12 196 (84.3%) had a normal cycle length (26-31 days), and only 78 of 12 196 (0.6%) reported an extreme cycle length (<21 days or ≥40 days or too irregular to estimate).^13^ Similarly, among women who reported that their cycles were always irregular or had no periods, only 295 of 2875 (10.3%) reported a normal cycle length, and 1788 of 2875 (62.2%) had an extreme cycle length.

### Ascertainment of Type 2 Diabetes

Self-reports of physician-diagnosed type 2 diabetes on follow-up questionnaires were confirmed with validated supplementary questionnaires. For type 2 diabetes cases reported before 1998, the diagnosis was confirmed according to the National Diabetes Data Group criteria.^14^ From 1998 onward, the American Diabetes Association diagnostic criteria were applied for type 2 diabetes identification.^15^ The validity of this ascertainment method has been previously documented. In a random sample of 62 type 2 diabetes cases confirmed by the supplementary questionnaire, 98.4% of cases (n = 61) were reconfirmed through medical record review.^16^ Moreover, among 200 participants without a prior report of diabetes, only 1 (0.5%) had an elevated fasting plasma glucose or plasma fructosamine level.^17^

### Covariates

Height and race/ethnicity were reported by participants at recruitment. Data on lifestyle factors and health-related characteristics were obtained at baseline and updated every 2 to 4 years. Dietary intake and alcohol intake were assessed every 4 years since 1991 using a validated, semiquantitative food frequency questionnaire. We computed the Alternate Healthy Eating Index (AHEI)–2010 as a summary measure of diet quality. Women with a BMI of 25 or more, current smokers, women in the bottom 60% of diet quality as ascertained with the AHEI-2010 score, and women who engaged in less than 150 minutes per week of moderate- to vigorous-intensity activity were considered to be in the high-risk group for each of these factors.^18,19^ For each lifestyle factor, participants received a score of 1 if they met the criterion for high risk or 0 otherwise. The overall unhealthy lifestyle score was the sum of each lifestyle factor.

### Statistical Analysis

Statistical analysis was performed from February 1 to December 30, 2019. The follow-up period was estimated from the date of return of the 1993 questionnaire until the diagnosis of type 2 diabetes, death, or the end of follow-up (June 30, 2017), whichever occurred first. The participants who were lost to follow-up or died before the diagnosis of type 2 diabetes were treated as censored observations in all analyses. Percentages are standardized to the age distribution of the study population. Cox proportional hazards regression models were separately constructed to estimate the hazard ratios (HRs) and 95% CIs for the associations of menstrual cycle regularity or length at different age ranges with the risk of type 2 diabetes while simultaneously adjusting for time-varying confounders and risk factors. The time scale for the analysis was months since the start of the current questionnaire cycle, which is equivalent to age in months. Because oral contraceptives (OCs) are associated with menstrual cycle characteristics and OCs are used to treat common ovulation disorders,^20^ women who used OCs for more than 2 months during each age range of interest were included in a separate exposure category. We also assessed the risk of type 2 diabetes according to the change in menstrual cycle patterns across the reproductive life span.

Multivariable Cox proportional hazards regression models were adjusted for age, age at menarche, race/ethnicity, and family history of type 2 diabetes as well as for time-varying potential confounders, including menopausal status, parity, household income, OC use, and alcohol consumption. The multivariable Cox proportional hazards regression models were further adjusted for time-varying BMI, physical activity, smoking status, and AHEI-2010 diet score. Information from the previous questionnaire was carried forward for missing data (<5% for any covariates); otherwise, a separate missing data category was created.

We estimated the HRs according to the joint categories of cycle regularity and length and examined their interaction. Given the strong associations of BMI with menstrual cycle dysfunction and type 2 diabetes,^21,22^ we further performed analyses stratified by time-varying BMI. Multiplicative interaction was estimated using the likelihood ratio test to evaluate whether the combined association of menstrual cycle dysfunction and unhealthy lifestyle was larger than the product of the estimated association of cycle dysfunction alone and unhealthy lifestyle alone.^23^ The additive interaction was assessed by calculating the relative excess risk due to interaction,^24^ which evaluated whether the combined association of 2 factors was larger than the summed individual association of cycle dysfunction and unhealthy lifestyle.^23^

Several sensitivity analyses were conducted. First, we excluded women aged 40 years or older in 1993 to avoid misclassification of women experiencing early signs of menopause. Second, we excluded women reporting no period or cycles longer than 50 days or too irregular to estimate to reduce misclassification. Third, we included women who provided partial data on menstrual cycle characteristics at the age ranges of 14 to 17 years, 18 to 22 years, and 29 to 46 years. Fourth, we excluded women reporting endometriosis, hirsutism, or uterine fibroids to test potential confounding by other gynecologic conditions. Fifth, we excluded women with type 2 diabetes that occurred before 1997 who were part of our previous study.^9^ Sixth, we did not carry forward the covariates with missing data. Seventh, we defined women with a BMI of 30 or more as the high-risk group. All data were analyzed using SAS, version 9.4 for UNIX (SAS Institute Inc), and statistical significance was set at a 2-tailed *P* < .05.

## Results

Among the 75 546 women in the study, at baseline, the mean (SD) age was 37.9 (4.6) years (range, 29.0-46.0 years). Compared with women who reported very regular cycles, women reporting irregular cycles or no period had a higher mean (SD) BMI (28.1 [7.8] vs 24.9 [5.3]) and mean (SD) total caloricintake (1825.5 [567.0] vs 1798.5 [543.4] kcal/d), as well as lower mean (SD) alcohol consumption (2.6 [6.5] vs 3.2 [6.1] g/d) and lower mean (SD) AHEI-2010 diet score (46.9 [10.8] vs 48.2 [10.8]) (Table 1). Women reporting always irregular cycles or no period were more likely than women who reported very regular cycles to be current smokers (302 of 2542 [11.9%] vs 4333 of 41 698 [10.3%]) and have a family history of diabetes (484 of 2542 [19.1%] vs 6599 of 41 698 [15.6%]). Similar results were observed among women reporting that their usual cycle length was 40 days or more compared with women with a normal cycle length between 26 and 31 days (eTable 2 in the Supplement).

**Table 1.** Age-Standardized Characteristics of the Study Population at Baseline by Menstrual Cycle Regularity Between the Ages of 29 and 46 Years (Nurses’ Health Study II, 1993-2017)

Characteristic	Menstrual cycle regularity^a^			
	Very regular (within 3-4 d of expected period) (n = 41 698)	Regular (within 5-7 d of expected period) (n = 17 871)	Usually irregular (n = 4321)	Always irregular or no period (n = 2542)
Age, mean (SD), y^b^	38.1 (4.4)	38.3 (4.5)	38.4 (5.0)	37.6 (4.9)
Age at menarche, mean (SD), y	12.4 (1.4)	12.5 (1.4)	12.5 (1.6)	12.6 (1.6)
White, No. (%)	40 079 (96.1)	17 047 (95.4)	4088 (94.6)	2422 (95.3)
Current smoker, No. (%)	4333 (10.3)	1954 (10.8)	489 (10.9)	302 (11.9)
Physical activity, mean (SD), h/wk	2.7 (3.8)	2.5 (3.5)	2.5 (3.8)	2.5 (4.0)
BMI, mean (SD)	24.9 (5.3)	25.1 (5.6)	26.6 (6.9)	28.1 (7.8)
Hirsutism, No. (%)	777 (1.8)	517 (2.9)	205 (4.8)	212 (8.5)
Endometriosis, No. (%)	1180 (4.5)	807 (4.5)	228 (5.3)	152 (5.9)
Uterine fibroid, No. (%)	3315 (7.8)	1553 (8.3)	425 (9.2)	247 (9.9)
Parity, mean (SD)	1.7 (1.2)	1.8 (1.2)	1.6 (1.2)	1.6 (1.3)
Family history of diabetes, No. (%)	6599 (15.6)	2990 (16.4)	744 (17.0)	484 (19.1)
Alcohol consumption, mean (SD), g/d	3.2 (6.1)	2.9 (5.8)	2.6 (5.2)	2.6 (6.5)
Total caloric intake, mean (SD), kcal/d	1798.5 (543.4)	1814.7 (547.3)	1802.7 (551.6)	1825.5 (567.0)
Alternate Healthy Eating Index score, mean (SD)	48.2 (10.8)	47.6 (10.8)	47.3 (10.7)	46.9 (10.8)

Abbreviation: BMI, body mass index (calculated as weight in kilograms divided by height in meters squared).

^a^ Percentages are standardized to the age distribution of the study population. Age-standardized characteristics of oral contraceptive users (n = 9144) were not shown.

^b^ Value is not age adjusted.

**Figure 1.**
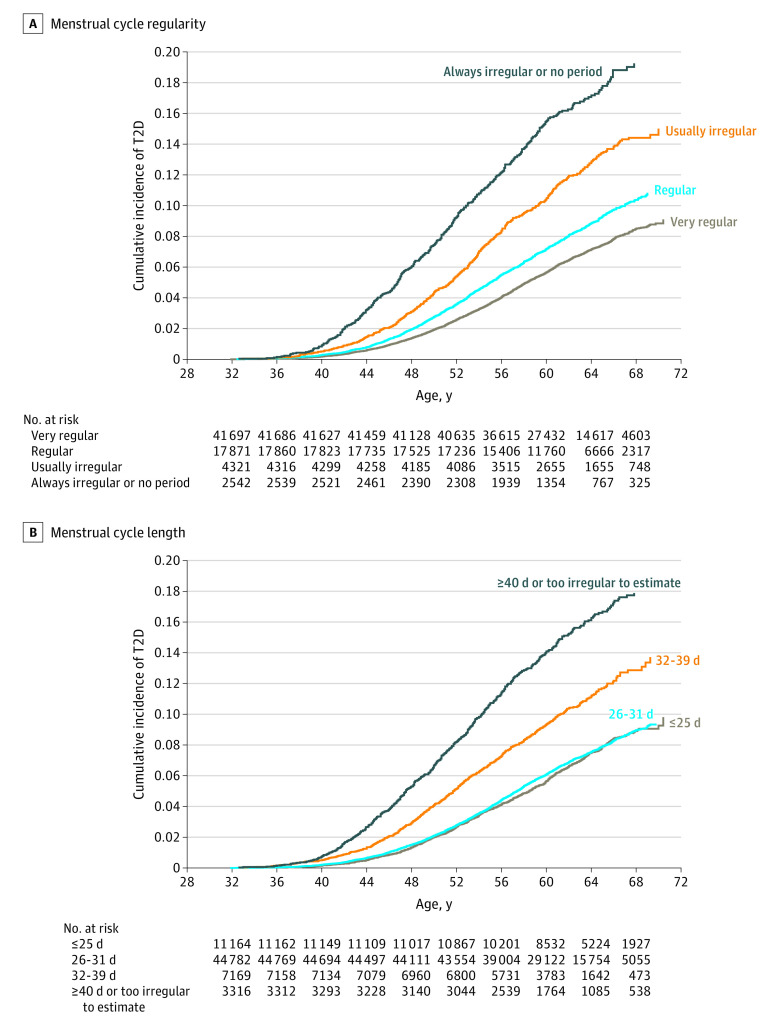
Crude Cumulative Incidence of Type 2 Diabetes (T2D) According to Menstrual Cycle Regularity and Length During Mid-Adulthood The cumulative incidence of T2D of oral contraceptive users (n = 9144) was not shown.

**Figure 2.**
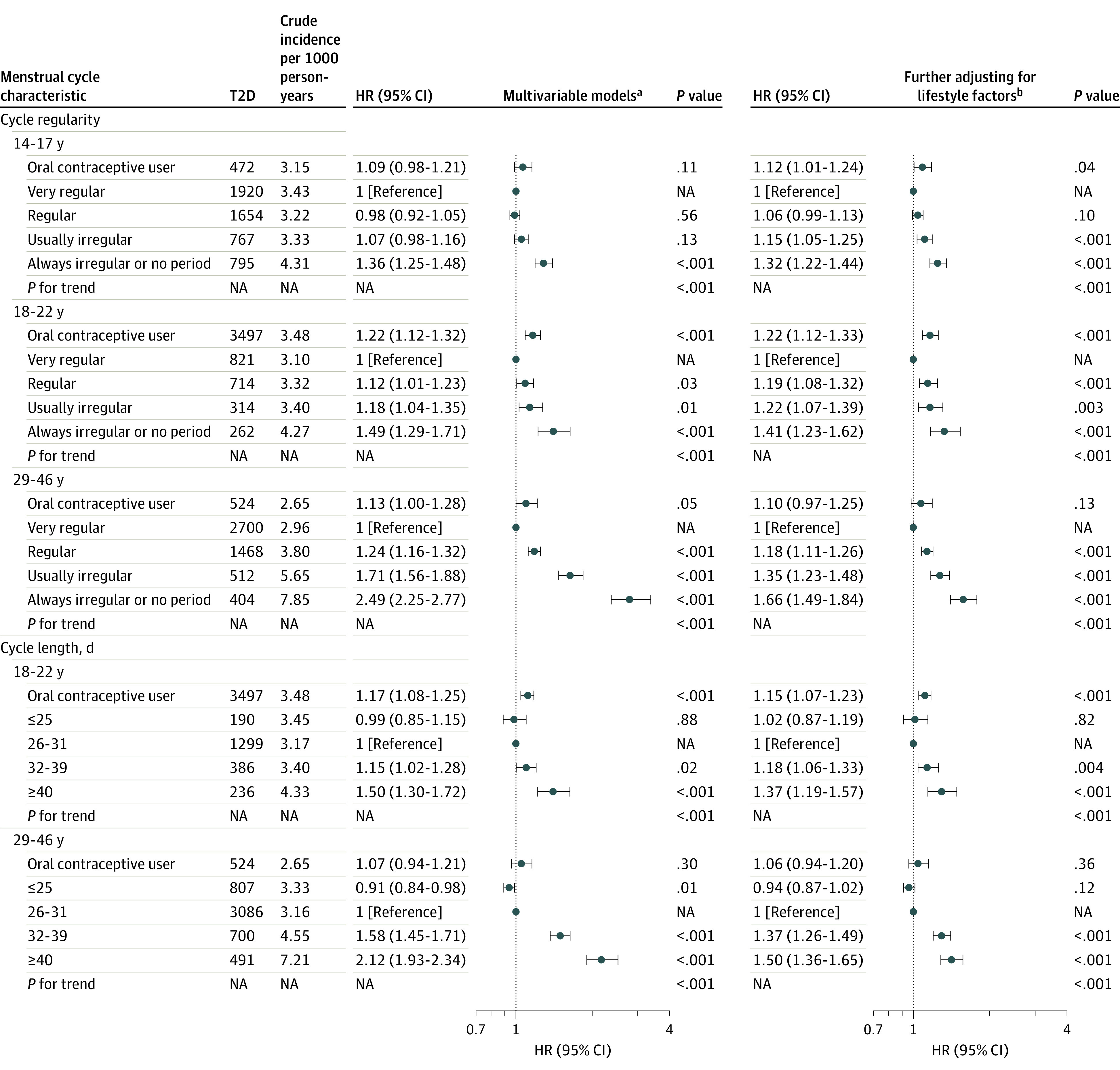
Adjusted Hazard Ratios (HRs) for Risk of Type 2 Diabetes (T2D) According to Menstrual Cycle Regularity and Length (Nurses’ Health Study II, 1993-2017) NA indicates not applicable. ^a^Models were adjusted for age (continuous), age at menarche (continuous), race/ethnicity (White, African American, Hispanic, or Asian), and family history of diabetes as well as for time-varying menopausal status (premenopausal; never, past, or current menopausal hormone use), parity (≤1, 2, or ≥3), household income (<$50 000, $50 000-$99 999, or≥$100 000), oral contraceptive use (never, past, or current), and alcohol consumption (0, 0.1-4.9, 5.0-9.9, 10.0-14.9, 15.0-29.9, or ≥30 g/d). ^b^Multivariable models were further adjusted for time-varying body mass index (calculated as weight in kilograms divided by height in meters squared; <23, 23-24.9, 25-29.9, 30-34.9, or ≥35), physical activity (0, 0.1-1.0, 1.1-3.4, 3.5-5.9, or ≥6 hours/week), smoking status (never smoker, former smoker, current smoker: 1-14, 15-24, or ≥25 cigarettes/d), and Alternate Healthy Eating Index diet quality score (quintiles). *P* value for trend was estimated by excluding oral contraceptive users.

We documented 5608 (7.4%) incident cases of type 2 diabetes during 1 639 485 person-years of follow-up. The crude cumulative incidence of type 2 diabetes was higher among women who reported irregular or long menstrual cycles than those reporting regular or short cycles (Figure 1). In the final multivariable models with adjustment for time-varying BMI and lifestyle risk factors, women reporting that their menstrual cycles were always irregular or had no period between the age ranges of 14 to 17 years, 18 to 22 years, and 29 to 46 years were, respectively, 32% (95% CI, 22%-44%), 41% (95% CI, 23%-62%), and 66% (95% CI, 49%-84%) more likely to develop type 2 diabetes than women reporting very regular cycles in the same age ranges (Figure 2). Similarly, women reporting that their usual cycle length was 40 days or more or too irregular to estimate between the age ranges of 18 to 22 years and 29 to 46 years were 37% (95% CI, 19%-57%) and 50% (95% CI, 36%-65%), respectively, more likely to develop type 2 diabetes during follow-up than women reporting a usual cycle length of 26 to 31 days in the same age ranges (Figure 2). There was no evidence of interaction between cycle regularity and length (eTable 3 in the Supplement). Women who used OCs between the age ranges of 14 to 17 years and 18 to 22 years had a higher risk of type 2 diabetes than women in the reference category of cycle length or regularity during the same age ranges (Figure 2).

When women were cross-classified according to menstrual cycle length and regularity across the reproductive life span, the risk of type 2 diabetes was strongest among women who consistently reported irregular cycles (adjusted HR, 1.55 [95% CI, 1.34-1.80]) and those whose cycle length changed from less than 32 days to 32 days or more (adjusted HR, 1.62 [95% CI, 1.39-1.88]) (Table 2). The associations of irregular and long menstrual cycles in mid-adulthood (29-46 years) with greater risk of type 2 diabetes persisted across all BMI categories (eTable 4 in the Supplement). Further analysis revealed an additive interaction of irregular and long menstrual cycles and overweight or obesity with the risk of type 2 diabetes, while there was no evidence of multiplicative interaction (Table 3). The additive interactions also persisted between irregular and long menstrual cycles and physical inactivity and low-quality diet. The relative excess risk of type 2 diabetes due to the interaction between irregular and long menstrual cycles and the overall unhealthy lifestyle score was 0.73 (95% CI, 0.57-0.89) and 0.68 (95% CI, 0.54-0.83), respectively.

**Table 2.** Adjusted Hazard Ratios for the Risk of Type 2 Diabetes According to Changes in Menstrual Cycle Characteristics Among 75 546 Premenopausal Women (Nurses’ Health Study II, 1993-2017)

Cycle characteristic at age 29-46 y	Type 2 diabetes, No.	Crude incidence, per 1000 person-years	Hazard ratio (95% CI)	
			Multivariable models^a^	Further adjusting for lifestyle factors^b^
**Change in regularity from 14-17 y to 29-46 y**				
Very regular or regular maintained	2915	3.24	1 [Reference]	1 [Reference]
Very regular or regular to usually or always irregular	350	6.63	1.65 (1.48-1.85)	1.28 (1.14-1.43)
Usually or always irregular to very regular or regular	932	3.24	1.05 (0.98-1.13)	1.12 (1.04-1.20)
Usually or always irregular maintained	478	6.36	2.05 (1.86-2.26)	1.54 (1.39-1.69)
Oral contraceptive users	933	2.89	1.12 (1.03-1.22)	1.09 (1.00-1.19)
**Change in regularity from 18-22 y to 29-46 y**				
Very regular or regular maintained	1260	3.07	1 [Reference]	1 [Reference]
Very regular or regular to usually or always irregular	146	7.24	1.84 (1.55-2.19)	1.34 (1.13-1.60)
Usually or always irregular to very regular or regular	304	3.00	1.02 (0.90-1.16)	1.08 (0.95-1.22)
Usually or always irregular maintained	210	6.17	2.13 (1.84-2.46)	1.55 (1.34-1.80)
Oral contraceptive users	3688	3.44	1.23 (1.14-1.32)	1.16 (1.08-1.25)
**Change in length from 18-22 y to 29-46 y**				
<32 d maintained	1148	2.97	1 [Reference]	1 [Reference]
<32 to ≥32 d	204	6.80	2.13 (1.83-2.47)	1.62 (1.39-1.88)
≥32 to <32 d	296	3.33	1.16 (1.02-1.32)	1.25 (1.10-1.42)
≥32 d maintained	272	4.45	1.75 (1.53-1.99)	1.44 (1.26-1.64)
Oral contraceptive users	3688	3.44	1.28 (1.19-1.38)	1.22 (1.13-1.31)

^a^ Models were adjusted for age (continuous), age at menarche (continuous), race/ethnicity (White, African American, Hispanic, or Asian), and family history of diabetes, as well as updated menopausal status (premenopausal; never, past, or current menopausal hormone use), parity (≤1, 2, or ≥3), household income (<$50 000, $50 000-$99 999, or ≥$100 000), oral contraceptive use (never, past, or current), and alcohol consumption (0, 0.1-4.9, 5.0-9.9, 10.0-14.9, 15.0-29.9, or ≥30 g/d).

^b^ Multivariable models were further adjusted for updated body mass index (calculated as weight in kilograms divided by height in meters squared; <23, 23-24.9, 25-29.9, 30-34.9, or ≥35), physical activity (0, 0.1-1.0, 1.1-3.4, 3.5-5.9, or ≥6 hours/week), smoking status (never smoker, former smoker, current smoker: 1-14, 15-24, or ≥25 cigarettes/d), and Alternate Healthy Eating Index diet quality score (quintiles).

**Table 3.** Additive Interaction of Menstrual Cycle Characteristics During Mid-Adulthood (29-46 Years) and Lifestyle Factors With Risk of Type 2 Diabetes Among Premenopausal Women (Nurses’ Health Study II, 1993-2017)^a^

Additive interaction	Estimations (95% CI)				
	BMI^b^	Smoking status^c^	Dietary quality^d^	Physical activity^e^	Overall unhealthy score^f^
**Interaction for cycle regularity**^**g**^					
Main effects					
Usually or always irregular or no period vs very regular or regular period	1.49 (1.05 to 2.12)	1.84 (1.68 to 2.01)	1.76 (1.52 to 2.02)	1.81 (1.55 to 2.11)	2.10 (1.66 to 2.64)
Unhealthy lifestyles	10.83 (9.51 to 12.34)	1.19 (1.12 to 1.27)	1.43 (1.33 to 1.53)	1.81 (1.69 to 1.95)	1.91 (1.85 to 1.97)
Joint effect	6.10 (4.64 to 7.57)	1.93 (1.82 to 2.05)	2.52 (2.42 to 2.62)	3.23 (3.13 to 3.33)	3.74 (3.56 to 3.91)
Relative excess risk due to interaction	17.43 (17.28 to 17.57)	−0.10 (−0.37 to 0.17)	0.34 (0.04 to 0.64)	0.61 (0.25 to 0.96)	0.73 (0.57 to 0.89)
*P* value for additive interaction	<.001	.47	.03	<.001	<.001
*P* value for multiplicative interaction	.68	.10	.94	.86	.08
**Interaction for cycle length**^**g**^					
Main effects					
Length ≥32 d vs <32 d	1.52 (1.13 to 2.06)	1.84 (1.70 to 2.00)	1.73 (1.52 to 1.96)	1.63 (1.41 to 1.87)	1.84 (1.50 to 2.27)
Unhealthy lifestyles	10.77 (9.41 to 12.32)	1.20 (1.13 to 1.29)	1.42 (1.32 to 1.53)	1.77 (1.64 to 1.90)	1.90 (1.84 to 1.97)
Joint effect	6.24 (4.89 to 7.59)	1.97 (1.86 to 2.08)	2.54 (2.45 to 2.63)	3.24 (3.15 to 3.33)	3.43 (3.27 to 3.59)
Relative excess risk due to interaction	17.53 (17.39 to 17.68)	−0.08 (−0.32 to 0.17)	0.39 (0.13 to 0.66)	0.85 (0.55 to 1.15)	0.68 (0.54 to 0.83)
*P* value for additive interaction	<.001	.55	.004	<.001	<.001
*P* value for multiplicative interaction	.67	.09	.66	.13	.53

Abbreviation: BMI, body mass index (calculated as weight in kilograms divided by height in meters squared).

^a^ Models were adjusted for age (continuous), age at menarche (continuous), race/ethnicity (White, African American, Hispanic, or Asian), and family history of diabetes, as well as updated menopausal status (premenopausal; never, past, or current menopausal hormone use), parity (≤1, 2, or ≥3), household income (<$50 000, $50 000-$99 999, or ≥$100 000), oral contraceptive use (never, past, or current), and alcohol consumption (0, 0.1-4.9, 5.0-9.9, 10.0-14.9, 15.0-29.9, or ≥30 g/d).

^b^ Tested using categorical variables (<25 vs ≥25).

^c^ Smoking status was tested using dichotomous variables (current vs noncurrent smokers).

^d^ Dietary quality was tested using dichotomous variables (bottom three-fifths vs upper two-fifths of Alternate Healthy Eating Index diet quality score).

^e^ Physical activity was tested using dichotomous variables (<30 vs ≥30 minutes/d at moderate intensity).

^f^ Unhealthy lifestyle scores were calculated by including current smoking, exercise less than 30 minutes/d at moderate intensity, diet in bottom three-fifths of Alternate Healthy Eating Index diet quality score, and BMI of 25 or more.

^g^ Oral contraceptive users (n = 9144) were not included in the analysis.

Multivariable Cox proportional hazards regression models showed similar results when we included the women who provided partial information on their menstrual cycle characteristics; when we excluded the women aged 40 years or older in 1993, those who reported no periods, those who reported a cycle greater than 50 days or too irregular to estimate, those with endometriosis, those with hirsutism, those with uterine fibroids, or those who received a diagnosis of type 2 diabetes before 1997; and when the covariates with missing data were not carried forward (eTable 5 in the Supplement). The results of interaction analysis were also substantially unchanged when we defined women with a BMI of 30 or more as the high-risk group (eTable 6 in the Supplement).

## Discussion

In this large, prospective cohort, women who experienced irregular or long menstrual cycles in adolescence and throughout adulthood were more likely to develop type 2 diabetes than women without menstrual cycle dysfunction. The risk was greatest among women who maintained irregular cycles across the reproductive life span and those whose cycle length changed from less than 32 days to 32 days or more. In addition, we found an additive interaction of menstrual cycle dysfunction and overweight and obesity, physical inactivity, and low-quality diet with risk of type 2 diabetes.

A disrupted hormonal environment is suspected to play a critical role in the association between menstrual cycle dysfunction and the risk of type 2 diabetes. Irregular and long menstrual cycles are strong indicators of hyperinsulinemia, which can synergize with pituitary gonadotropins to stimulate androgen production in ovarian theca cells, further exacerbating insulin resistance and increasing the risk of type 2 diabetes.^25^ Hyperinsulinemia can also inhibit sex hormone–binding globulin excretion, leading to higher serum concentrations of unbound testosterone.^26^ This hormonal milieu has been hypothesized to play a critical role in the cause of type 2 diabetes.^27^ Furthermore, menstrual cycle disorders are also associated with dysregulated inflammatory processes,^28^ which may also be involved in the development of type 2 diabetes. Results from a systematic review also suggested that women with polycystic ovary syndrome, a common endocrine disorder characterized by ovarian dysfunction—including long or irregular cycles—and excess androgens, had higher circulating concentrations of multiple oxidative stress markers, such as homocysteine, malondialdehyde, asymmetric dimethylarginine, and superoxide dismutase.^29^

Although some studies have reported no association between long or irregular cycles and risk of type 2 diabetes,^6,8^ our findings are in agreement with the preponderance of the evidence to date. Consistent with an earlier report from this cohort, Solomon and colleagues^9^ showed that women with long and highly irregular cycles at the ages of 18 to 22 years had an elevated risk of type 2 diabetes after 6 years of follow-up. Our results are also in agreement with another study conducted among 124 379 postmenopausal women aged 50 to 79 years, which found that women reporting irregular menstrual cycles during most of their life experienced a greater risk of type 2 diabetes.^7^ Similarly, several observational studies have also identified associations of irregular and long menstrual cycles with a higher risk of insulin resistance^30^ and gestational diabetes.^31^

Although it is clear that obesity and key lifestyle factors are important risk factors for type 2 diabetes,^10^ the joint association of menstrual cycle dysfunction and unhealthy lifestyle with the risk of type 2 diabetes has not been evaluated, to our knowledge. In this study, the excess risk of menstrual cycle dysfunction combined with overweight or obesity was higher than the summed risk associated with each individual factor. However, this association was not completely dependent on BMI, and stratified analyses showed a greater risk of type 2 diabetes among women with long and irregular cycles across all BMI categories. Both findings are equally important. First, the presence of the association across the BMI spectrum highlights the importance of including cycle dysfunction as a risk factor for type 2 diabetes, even among women with normal BMI. In addition, the significant additive interaction between cycle characteristics and BMI suggests that the adverse hormonal and inflammatory risk profile observed among women with long or irregular cycles is further exacerbated by overweight and obesity,^21^ making weight management strategies particularly important for women presenting with cycle dysfunction. Meanwhile, we also found a significant additive interaction of unhealthy menstrual cycle patterns and physical inactivity and low-quality diet with risk of type 2 diabetes, emphasizing the importance of maintaining an overall healthy lifestyle in preventing type 2 diabetes.

### Strengths and Limitations

This study has some strengths. Most of the previous studies asked the participants to recall menstrual cycle patterns in the distant past and roughly characterized cycle length and regularity as a dichotomy (eg, regular vs irregular),^6,7,8^ which may have resulted in insufficiently precise results. Previous studies also did not have sufficient sample sizes to address the role of concomitant OC use at the time of assessment of menstrual cycle characteristics. Because OCs change menstrual cycle characteristics and are often used as a first-line treatment for women presenting with menstrual cycle disorders, hyperandrogenism, or frank polycystic ovary syndrome, there was probably estimation bias owing to the “noise” of OC use. Categorizing person-time during which women were OC users into a separate exposure group allowed us to obtain estimates that are not only completely independent of OC use but also separate from the reference group of women who may be using OCs to manage abnormal cycles. We found that OC use in adolescence and early adulthood was associated with a greater risk of type 2 diabetes, supporting some previous studies reporting an association between OC use and insulin resistance and diabetes.^32,33^ Additional strengths of this study include its long-term follow-up period, large sample size, measures of various confounders and lifestyle factors, and collection of menstrual cycle characteristics at different age ranges.

Our study also has some limitations. First, while several authors have assessed the validity of self-reported menstrual cycle characteristics,^3,12^ measurement error cannot be fully excluded. However, given that the measurement error in the assessment of menstrual cycle characteristics is expected to be nondifferential to type 2 diabetes, the associations were more likely to be attenuated toward the null. In support of this notion, we found a greater risk of type 2 diabetes among women reporting long or irregular cycles later in life, which might be a result of diminished recall accuracy for earlier age ranges.^34^ Second, many women did not fully report their menstrual cycle characteristics across the reproductive life span, which may have resulted in selection bias. However, we observed similar baseline characteristics between included and excluded participants owing to missing exposure data. In addition, our findings were substantially unchanged when we included women who provided partial data on cycle characteristics. Third, study participants had a relatively homogeneous racial/ethnic and educational background, potentially hampering the generalizability of our findings.

## Conclusions

In this cohort study of US female nurses participating in the Nurses’ Health Study II, irregular and long menstrual cycles throughout life were associated with a greater long-term risk of type 2 diabetes, particularly among women who adopted unhealthy lifestyles. These results highlight the need for women’s health care professionals to consider menstrual cycle characteristics across the reproductive life span as an independent sign when evaluating the metabolic risk of their patients and point to potential lifestyle interventions to prevent the development of type 2 diabetes in women with menstrual cycle dysfunction.

## Notes

Supplement. **eTable 1.** Age-Standardized Characteristics of Included vs Excluded Women at Baseline in 1993 (The Nurses’ Health Study II, 1993-2017) **eTable 2.** Age-Standardized Characteristics of the Study Population at Baseline by Cycle Length Between Ages of 29-46 Years (The Nurses’ Health Study II, 1993-2017) **eTable 3.** Adjusted Hazard Ratios (HRs) and 95% Confidence Intervals (CI) for the Risk of T2D According to Joint Categories of Menstrual Cycle Regularity and Length Among 75 546 Premenopausal Women (The Nurses’ Health Study II, 1993-2017) **eTable 4.** Adjusted Hazard Ratios (HRs) and 95% Confidence Intervals (CIs) for Risk of T2D According to Midadulthood (age 29-46 Years) Cycle Regularity and Length Among 75 546 Premenopausal Women, Stratified by Updated BMI (The Nurses’ Health Study II, 1993-2017) **eTable 5.** Sensitivity Analysis for the Association of Menstrual Cycle Regularity (Age 14-17, 18-22, and 18-48 Years) and Length (Age 18-22 and 29-46 Years) With the Risk of T2D (The Nurses’ Health Study II, 1993-2017) **eTable 6.** Adjusted Attributing Effects to Additive Interaction Between Menstrual Cycle Characteristics During Mid-adulthood (Age 29-46 Years) and Lifestyle Factors on Risk of T2D Among Premenopausal Women (The Nurses’ Health Study II, 1993-2017)
