# Errors in Figure 2

**DOI:** 10.1001/jamanetworkopen.2020.34976

**Published:** 2021-01-11

In the Original Investigation titled “Associations of Menstrual Cycle Characteristics Across the Reproductive Life Span and Lifestyle Factors With Risk of Type 2 Diabetes,”^1^ published online December 21, 2020, the age range of 28 to 46 years should be changed to 29 to 46 years in 2 places in Figure 2. This article has been corrected.
