# Supplementary material for: Associations of Menstrual Cycle Characteristics Across the Reproductive Life Span and Lifestyle Factors With Risk of Type 2 Diabetes
Source: JAMA Netw Open. 2020 Dec 21;3(12):e2027928. doi: 10.1001/jamanetworkopen.2020.27928 (PMC7753904; doi:10.1001/jamanetworkopen.2020.27928)
Supplement: Supplement. — eTable 1. Age-Standardized Characteristics of Included vs Excluded Women at Baseline in 1993 (The Nurses’ Health Study II, 1993-2017) eTable 2. Age-Standardized Characteristics of the Study Population at Baseline by Cycle Length Between Ages of 29-46 Years (The Nurses’ Health Study II, 1993-2017) eTable 3. Adjusted Hazard Ratios (HRs) and 95% Confidence Intervals (CI) for the Risk of T2D According to Joint Categories of Menstrual Cycle Regularity and Length Among 75 546 Premenopausal Women (The Nurses’ Health Study II, 1993-2017) eTable 4. Adjusted Hazard Ratios (HRs) and 95% Confidence Intervals (CIs) for Risk of T2D According to Midadulthood (age 29-46 Years) Cycle Regularity and Length Among 75 546 Premenopausal Women, Stratified by Updated BMI (The Nurses’ Health Study II, 1993-2017) eTable 5. Sensitivity Analysis for the Association of Menstrual Cycle Regularity (Age 14-17, 18-22, and 18-48 Years) and Length (Age 18-22 and 29-46 Years) With the Risk of T2D (The Nurses’ Health Study II, 1993-2017) eTable 6. Adjusted Attributing Effects to Additive Interaction Between Menstrual Cycle Characteristics During Mid-adulthood (Age 29-46 Years) and Lifestyle Factors on Risk of T2D Among Premenopausal Women (The Nurses’ Health Study II, 1993-2017) [file jamanetwopen-e2027928-s001.pdf]

Supplementary Online Content

Wang YX, Shan Z, Arvizu M, et al. Associations of menstrual cycle characteristics across the reproductive life span and lifestyle factors with risk of type 2 diabetes. *JAMA Netw Open.* 2020;3(12):e2027928. doi:10.1001/jamanetworkopen.2020.27928

eTable 1. Age-Standardized Characteristics of Included vs Excluded Women at Baseline in 1993 (The Nurses' Health Study II, 1993-2017)
eTable 2. Age-Standardized Characteristics of the Study Population at Baseline by Cycle Length Between Ages of 29-46 Years (The Nurses' Health Study II, 1993-2017)
eTable 3. Adjusted Hazard Ratios (HRs) and 95% Confidence Intervals (CIs) for the Risk of T2D According to Joint Categories of Menstrual Cycle Regularity and Length Among 75 546 Premenopausal Women (The Nurses' Health Study II, 1993-2017)
eTable 4. Adjusted Hazard Ratios (HRs) and 95% Confidence Intervals (CIs) for Risk of T2D According to Midadulthood (Age 29-46 Years) Cycle Regularity and Length Among 75 546 Premenopausal Women, Stratified by Updated BMI (The Nurses' Health Study II, 1993-2017)
eTable 5. Sensitivity Analysis for the Association of Menstrual Cycle Regularity (Age 14-17, 18-22, and 18-48 Years) and Length (Age 18-22 and 29-46 Years) With the Risk of T2D (The Nurses' Health Study II, 1993-2017)
eTable 6. Adjusted Attributing Effects to Additive Interaction Between Menstrual Cycle Characteristics During Mid-adulthood (Age 29-46 Years) and Lifestyle Factors on Risk of T2D Among Premenopausal Women (The Nurses' Health Study II, 1993-2017)

This supplementary material has been provided by the authors to give readers additional information about their work.

© 2020 Wang YX et al. *JAMA Network Open.*

eTable 1. Age-Standardized Characteristics of Included vs Excluded Women at Baseline in 1993 (The Nurses' Health Study II, 1993-2017)

Characteristics	Included[b]	Excluded due to missing data on exposure[c]	Excluded due to missing data on lifestyle factors[d]
No.	75 546	27 022	2419
Age, mean (SD), year[a]	37.7 (4.6)	38.7 (4.7)	37.2 (4.5)
Age at menarche, mean (SD), year	12.4 (1.4)	12.4 (1.5)	12.4 (1.5)
White, No (%)	72 350 (95.8)	24 673 (91.4)	2168 (89.4)
Current smoker, No (%)	7841 (10.4)	3574 (13.1)	288 (12.5)
Physical activity, mean (SD), hour/week	2.6 (3.8)	2.9 (4.6)	3.2 (5.3)
BMI, mean (SD), kg/m^2	25.1 (5.6)	25.3 (5.6)	25.6 (5.7)
Hirsutism, No (%)	1919 (2.6)	523 (1.9)	38 (1.7)
Endometriosis, No (%)	3479 (4.6)	1132 (4.1)	114 (4.7)
Uterine Fibroid, No (%)	5967 (8.1)	2877 (9.8)	195 (8.6)
Parity	1.7 (1.2)	1.6 (1.2)	1.7 (1.3)
Family history of diabetes, No (%)	12067 (16.1)	4693 (16.9)	419 (17.8)
Alcohol consumption, mean (SD), g/day	3.1 (6)	3.1 (6.3)	3.2 (7.4)
Total calories intake, mean (SD), kcal/day	1795.6 (545.3)	1777 (558.3)	1825.2 (498.8)
Alternative Healthy Eating Index, mean (SD)	48.1 (10.8)	48.3 (10.9)	48.9 (11.1)

Values are means (SD) for continuous variables and percentages for categorical variables and are standardized to the age distribution of the study population. [a]Value is not age-adjusted. [b]Women with complete lifestyle factors and menstrual cycle characteristics who were included in our analysis. [c]Women who were excluded from the analysis because of missing data on menstrual cycle characteristics at ages of 14-17, 18-22, or 29-46 years. [d]Women who were excluded from the analysis because of missing data on smoking, body mass index, diet, or physical activity at baseline and follow-ups.

© 2020 Wang YX et al. *JAMA Network Open.*

eTable 2. Age-Standardized Characteristics of the Study Population at Baseline by Cycle Length Between Ages of 29-46 Years (The Nurses' Health Study II, 1993-2017)

Characteristics	Cycle length[b]			
	**≤25 days**	**26-31 days**	**32-39 days**	**≥40 days or too irregular to estimate**
No.	11 164	44 783	7169	3316
Age, mean (SD), year[a]	39.4 (4.2)	38.1 (4.4)	36.8 (4.4)	37.8 (5.1)
Age at menarche, mean (SD), year	12.3 (1.4)	12.4 (1.4)	12.6 (1.5)	12.6 (1.6)
White, No (%)	10 632 (95.3)	42 994 (96.0)	6869 (95.8)	3141 (94.7)
Current smoker, No (%)	1615 (14.7)	4534 (10.1)	575 (8.3)	354 (10.5)
Physical activity, mean (SD), hour/week	2.7 (4.0)	2.6 (3.7)	2.5 (3.6)	2.4 (3.6)
BMI, mean (SD), kg/m^2	24.8 (5.3)	25.0 (5.4)	26.0 (6.4)	27.9 (7.7)
Hirsutism, No (%)	250 (2.1)	916 (2.0)	293 (4.3)	252 (8.0)
Endometriosis, No (%)	549 (5.0)	2070 (4.6)	301 (4.1)	147 (4.2)
Uterine Fibroid, No (%)	1125 (8.9)	3617 (7.9)	518 (7.8)	280 (8.3)
Parity	1.7 (1.2)	1.7 (1.2)	1.8 (1.2)	1.6 (1.3)
Family history of diabetes, No (%)	1904 (16.2)	7167 (15.8)	1153 (16.6)	593 (18.0)
Alcohol consumption, mean (SD), g/day	3.0 (5.7)	3.2 (6.1)	2.7 (5.5)	2.6 (5.6)
Total calories intake, mean (SD), kcal/day	1784.7 (553.7)	1803.1 (543.6)	1828.1 (544.9)	1827.0 (557.3)
Alternative Healthy Eating Index, mean (SD)	47.9 (10.8)	48.0 (10.8)	47.8 (10.9)	47.2 (10.7)

Values are means (SD) for continuous variables and percentages for categorical variables and are standardized to the age distribution of the study population. [a] Value is not age-adjusted. [b]Age-standardized characteristics of oral contraceptive users (n=9,144) were not shown.

© 2020 Wang YX et al. *JAMA Network Open.*

eTable 3. Adjusted Hazard Ratios (HRs) and 95% Confidence Intervals (CIs) for the Risk of T2D According to Joint Categories of Menstrual Cycle Regularity and Length Among 75 546 Premenopausal Women (The Nurses' Health Study II, 1993-2017)[a]

Regularity	Length	T2D/PY	Crude incidence, per 1,000 PY	HRs (95% CI)[a]
**18-22y**				
Oral contraceptive users	OC users	3497	3.48	1.16 (1.08-1.24)
Very regular or regular	<32 day	1336	3.17	1 [Reference]
	≥32 day	199	3.43	1.22 (1.05-1.42)
Irregular or no cycles	<32 day	153	3.51	1.12 (0.95-1.33)
	≥32 day	423	3.84	1.28 (1.14-1.43)
p for multiplicative interaction				.62
RERI (95% CI)				-0.05 (-0.34-0.23)
p for additive interaction				.70
**29-46y**				
Oral contraceptive users	OC users	524	2.65	1.08 (0.96-1.23)
Very regular or regular	<32 days	3659	3.12	1 [Reference]
	≥32 days	509	4.06	1.32 (1.21-1.45)
Irregular or no cycles	<32 days	234	5.12	1.16 (1.01, 1.32)
	≥32 days	682	7.07	1.56 (1.44-1.69)
p for multiplicative interaction				.81
RERI (95% CI)				0.08 (-0.14-0.31)
p for additive interaction				.47

[a]Models were adjusted for age (continuous), age at menarche (continuous), race/ethnicity (White, African-American, Hispanic, or Asian), and family history of diabetes, as well as time-varying menopausal status (premenopausal, never, past, or current menopausal hormone use), parity (≤1, 2, or ≥3), household income (<\$50000, \$50000–\$99999, or≥\$100000), oral contraceptive use (never, past, or current), alcohol consumption (0, 0.1-4.9, 5.0-9.9, 10.0-14.9, 15.0-29.9, or ≥30 g/day), BMI (<23, 23-24.9, 25-29.9, 30-34.9, or ≥35 kg/m^2), physical activity (0, 0.1-1.0, 1.1-3.4, 3.5-5.9, or ≥6 hours/week), smoking status (never smoker, former smoker, current smoker: 1-14, 15-24, or ≥25 cigarettes/day), and Alternative Healthy Eating Index diet quality score (quintiles).

© 2020 Wang YX et al. *JAMA Network Open.*

eTable 4. Adjusted Hazard Ratios (HRs) and 95% Confidence Intervals (CIs) for Risk of T2D According to Midadulthood (age 29-46 years) Cycle Regularity and Length Among 75 546 Premenopausal Women, Stratified by Updated BMI (The Nurses' Health Study II, 1993-2017)[a]

Menstrual cycle characteristics	Category of updated BMI		
	**18.5-24.9 kg/m^2 (n of T2D case=320)**	**25-29.9 kg/m^2 (n of T2D case=1066)**	**≥30 kg/m^2 (n of T2D case= 4215)**
**Cycle regularity**			
Oral contraceptive users	1.29 (0.80-2.05)	1.01 (0.76-1.33)	1.10 (0.95-1.27)
Very regular	1 [Reference]	1 [Reference]	1 [Reference]
Regular	1.07 (0.82-1.40)	1.06 (0.91-1.22)	1.21 (1.12-1.30)
Usually irregular	1.46 (0.95-2.25)	1.36 (1.08-1.71)	1.32 (1.18-1.47)
Always irregular/no period	1.57 (0.87-2.83)	1.44 (1.07-1.94)	1.60 (1.43-1.80)
P for trend[b]	.06	.002	<.001
**Cycle length**			
Oral contraceptive users	1.33 (0.83-2.13)	1.00 (0.75-1.32)	1.05 (0.90-1.21)
≤25 days	1.14 (0.84-1.55)	0.91 (0.77-1.08)	0.94 (0.86-1.03)
26-31 days	1 [Reference]	1 [Reference]	1 [Reference]
32-39 days	1.57 (1.10-2.25)	1.31 (1.06-1.61)	1.33 (1.21-1.46)
≥40d/too irregular to estimate	1.42 (0.83-2.42)	1.51 (1.17-1.94)	1.42 (1.28-1.58)
P for trend[b]	.18	<.001	<.001

[a]Models were adjusted for age (continuous), age at menarche (continuous), race/ethnicity (White, African-American, Hispanic, or Asian), and family history of diabetes, as well as time-varying menopausal status (premenopausal, never, past, or current menopausal hormone use), parity (≤1, 2, or ≥3), household income (<$50000, $50000–$99999, or≥$100000), oral contraceptive use (never, past, or current), alcohol consumption (0, 0.1-4.9, 5.0-9.9, 10.0-14.9, 15.0-29.9, or ≥30 g/day), BMI (<23, 23-24.9, 25-29.9, 30-34.9, or ≥35 kg/m^2), physical activity (0, 0.1-1.0, 1.1-3.4, 3.5-5.9, or ≥6 hours/week), smoking status (never smoker, former smoker, current smoker: 1-14, 15-24, or ≥25 cigarettes/day), and Alternative Healthy Eating Index diet quality score (quintiles). [b]*P* for trend was estimated by excluding oral contraceptive users.

© 2020 Wang YX et al. *JAMA Network Open.*

eTable 5. Sensitivity Analysis for the Association of Menstrual cycle Regularity (Age 14-17, 18-22, and 18-48 years) and Length (Age 18-22 and 29-46 years) With the Risk of T2D (The Nurses' Health Study II, 1993-2017)[a]

Menstrual cycle characteristics	Excluding women aged ≥40 y in 1993		Excluding women reporting no periods or too irregular to estimate		Excluding T2D cases diagnosed before 1997		Excluding women reporting hirsutism, endometriosis, or uterine fibroids		Including women reporting partial menstrual cycle data		Missing data was not carried forward	
	T2D	HRs (95% CI)	T2D	HRs (95% CI)	T2D	HRs (95% CI)	T2D	HRs (95% CI)	T2D	HRs (95% CI)	T2D	HRs (95% CI)
**Cycle regularity**												
**14-17y**												
Oral contraceptive users	337	1.02 (0.90-1.15)	472	1.12 (1.01-1.24)	445	1.12 (1.01-1.25)	274	1.06 (0.93-1.21)	652	1.10 (1.01-1.21)	472	1.10 (0.99-1.22)
Very regular	959	1 [Reference]	1920	1 [Reference]	1796	1 [Reference]	1204	1 [Reference]	2464	1 [Reference]	1920	1 [Reference]
Regular	816	1.03 (0.94-1.14)	1654	1.06 (0.99-1.13)	1567	1.07 (1.00-1.14)	1053	1.06 (0.97-1.15)	2076	1.03 (0.97-1.09)	1654	1.05 (0.98-1.12)
Usually irregular	378	1.10 (0.98-1.24)	767	1.15 (1.06-1.25)	721	1.15 (1.06-1.26)	485	1.18 (1.06-1.32)	995	1.15 (1.07-1.24)	767	1.14 (1.04-1.24)
Always irregular/no period	408	1.33 (1.19-1.50)	775	1.31 (1.21-1.43)	731	1.30 (1.19-1.42)	456	1.29 (1.15-1.44)	1007	1.31 (1.21-1.41)	795	1.32 (1.21-1.43)
P for trend[b]		<.001		<.001		<.001		<.001		<.001		<.001
**18-22y**												
Oral contraceptive users	1770	1.25 (1.11-1.40)	3497	1.22 (1.12-1.33)	3284	1.21 (1.11-1.32)	2156	1.24 (1.11-1.38)	4451	1.18 (1.09-1.27)	3497	1.22 (1.12-1.32)
Very regular	448	1 [Reference]	821	1 [Reference]	774	1 [Reference]	511	1 [Reference]	1029	1 [Reference]	821	1 [Reference]
Regular	354	1.12 (0.97-1.29)	714	1.19 (1.08-1.32)	676	1.20 (1.08-1.33)	459	1.23 (1.09-1.40)	862	1.13 (1.03-1.24)	714	1.18 (1.07-1.30)
Usually irregular	183	1.28 (1.07-1.52)	314	1.22 (1.07-1.39)	294	1.21 (1.06-1.39)	197	1.31 (1.11-1.55)	404	1.23 (1.10-1.38)	314	1.22 (1.07-1.39)
Always irregular/no period	143	1.32 (1.09-1.60)	259	1.41 (1.23-1.62)	232	1.33 (1.15-1.54)	149	1.33 (1.11-1.60)	327	1.36 (1.20-1.55)	262	1.40 (1.22-1.61)
P for trend[b]		<.001		<.001		<.001		<.001		<.001		<.001
**29-46y**												
Oral contraceptive users	435	1.16 (1.00-1.34)	524	1.10 (0.97-1.25)	500	1.09 (0.96-1.24)	343	1.14 (0.97-1.34)	550	1.09 (0.96-1.23)	524	1.10 (0.97-1.25)
Very regular	1282	1 [Reference]	2700	1 [Reference]	2543	1 [Reference]	1665	1 [Reference]	2791	1 [Reference]	2700	1 [Reference]
Regular	699	1.28 (1.16-1.40)	1468	1.18 (1.11-1.26)	1383	1.18 (1.11-1.26)	910	1.21 (1.11-1.31)	1516	1.18 (1.11-1.25)	1468	1.18 (1.11-1.26)
Usually irregular	249	1.56 (1.36-1.79)	512	1.35 (1.23-1.48)	471	1.34 (1.21-1.48)	305	1.36 (1.20-1.54)	535	1.36 (1.24-1.49)	512	1.38 (1.26-1.52)
Always irregular/no period	233	1.97 (1.71-2.27)	365	1.66 (1.49-1.86)	363	1.62 (1.45-1.81)	249	1.76 (1.53-2.01)	822	1.53 (1.41-1.66)	404	1.71 (1.54-1.91)

© 2020 Wang YX et al. *JAMA Network Open.*

P for trend[b]		<.001		<.001		<.001		<.001		<.001		<.001
**Cycle length**												
**18-22y**												
Oral contraceptive users	1770	1.22 (1.10-1.34)	3497	1.14 (1.06-1.23)	3284	1.13 (1.05-1.22)	2156	1.15 (1.05-1.26)	4584	1.13 (1.06-1.20)	3497	1.14 (1.06-1.23)
≤25 days	104	1.08 (0.88-1.33)	190	1.02 (0.87-1.19)	181	1.03 (0.88-1.21)	119	1.02 (0.84-1.24)	252	1.03 (0.90-1.17)	190	1.00 (0.86-1.17)
26-31 days	673	1 [Reference]	1299	1 [Reference]	1227	1 [Reference]	810	1 [Reference]	1630	1 [Reference]	1299	1 [Reference]
32-39 days	211	1.18 (1.01-1.38)	386	1.18 (1.06-1.33)	355	1.15 (1.02-1.30)	246	1.22 (1.05-1.40)	469	1.17 (1.06-1.30)	386	1.18 (1.05-1.32)
≥40 days /too irregular to estimate	140	1.50 (1.25-1.80)	58	1.21 (0.93-1.58)	213	1.31 (1.14-1.52)	141	1.33 (1.11-1.59)	295	1.35 (1.19-1.53)	236	1.37 (1.19-1.57)
P for trend[b]		<.001		.007		<.001		<.001		<.001		<.001
**29-46y**												
Oral contraceptive users	435	1.10 (0.95-1.27)	524	1.08 (0.95-1.23)	500	1.05 (0.92-1.19)	343	1.09 (0.93-1.28)	550	1.06 (0.93-1.19)	524	1.06 (0.93-1.20)
≤25 days	325	1.02 (0.90-1.15)	807	0.93 (0.86-1.01)	754	0.93 (0.86-1.01)	495	0.97 (0.88-1.07)	833	0.94 (0.87-1.01)	807	0.93 (0.86-1.01)
26-31 days	1455	1 [Reference]	3086	1 [Reference]	2912	1 [Reference]	1906	1 [Reference]	3187	1 [Reference]	3086	1 [Reference]
32-39 days	415	1.40 (1.25-1.56)	700	1.38 (1.27-1.50)	651	1.36 (1.25-1.48)	417	1.33 (1.19-1.47)	719	1.36 (1.25-1.47)	700	1.38 (1.27-1.50)
≥40 days /too irregular to estimate	268	1.81 (1.58-2.06)	89	1.43 (1.16-1.77)	443	1.46 (1.32-1.62)	311	1.52 (1.35-1.72)	503	1.48 (1.35-1.63)	491	1.54 (1.40-1.70)
P for trend[b]		<.001		<.001		<.001		<.001		<.001		<.001

[a]Models were adjusted for age (continuous), age at menarche (continuous), race/ethnicity (White, African-American, Hispanic, or Asian), and family history of diabetes, as well as time-varying menopausal status (premenopausal, never, past, or current menopausal hormone use), parity (≤1, 2, or ≥3), household income (<$50000, $50000–$99999, or≥$100000), oral contraceptive use (never, past, or current), alcohol consumption (0, 0.1-4.9, 5.0-9.9, 10.0-14.9, 15.0-29.9, or ≥30 g/day), BMI (<23, 23-24.9, 25-29.9, 30-34.9, or ≥35 kg/m^2), physical activity (0, 0.1-1.0, 1.1-3.4, 3.5-5.9, or ≥6 hours/week), smoking status (never smoker, former smoker, current smoker: 1-14, 15-24, or ≥25 cigarettes/day), and Alternative Healthy Eating Index diet quality score (quintiles). [b]*P* for trend was estimated by excluding oral contraceptive users.

© 2020 Wang YX et al. *JAMA Network Open.*

eTable 6. Adjusted attributing effects to additive interaction between menstrual cycle characteristics during mid-adulthood (age 29-46 years) and lifestyle factors on risk of T2D among premenopausal women (The Nurses' Health Study II, 1993-2017).[a]

Attributing effects	Estimations (95% CI)	
	BMI[b]	Overall unhealthy score[c]
**Interaction for cycle regularity**		
Main effects		
Usually or always irregular/no period vs. very regular or regular	1.50 (1.27, 1.76)	1.94 (1.57, 2.39)
Unhealthy lifestyles	7.19 (6.69, 7.73)	2.07 (2.01, 2.14)
Joint effect	10.61 (10.52, 10.71)	3.79 (3.63, 3.94)
RERI	2.92 (2.07, 3.78)	0.78 (0.60, 0.96)
P for additive interaction	<.001	<.001
P for multiplicative interaction	.89	.13
**Interaction for cycle length**		
Main effects		
Length ≥32 days vs. <32 days	1.46 (1.26, 1.68)	1.75 (1.45, 2.11)
Unhealthy lifestyles	7.08 (6.57, 7.63)	2.06 (1.99, 2.13)
Joint effect	10.82 (10.73, 10.91)	3.55 (3.40, 3.69)
RERI	3.28 (2.50, 4.05)	0.74 (0.58, 0.90)
P for additive interaction	<.001	<.001
P for multiplicative interaction	.58	.66

[a]Models were adjusted for age (continuous), age at menarche (continuous), ethnicity (White, African-American, Hispanic, or Asian), and family history of diabetes, as well as updated menopausal status (premenopausal, never, past, or current menopausal hormone use), parity (≤1, 2, or ≥3), household income (<$50000, $50000–$99999, or ⩾ $100000), oral contraceptive use (never, past, or current), and alcohol consumption (0, 0.1-4.9, 5.0-9.9, 10.0-14.9, 15.0-29.9, or ≥30 g/day). [b]BMI was tested using categorical variables (<30 vs. ≥30 kg/m^2). [c]Unhealthy lifestyle score was calculated by including current smoking, exercise <30 min/day at moderate intensity, diet in bottom three-fifths of Alternative Healthy Eating Index diet quality score, and BMI ≥30 kg/m^2.

© 2020 Wang YX et al. *JAMA Network Open.*
